# Generative AI costs in large healthcare systems, an example in revenue cycle

**DOI:** 10.1038/s41746-025-01971-x

**Published:** 2025-09-30

**Authors:** Michael L. Burns, Ssu-Ying Chen, Chu-An Tsai, John Vandervest, Balaji Pandian, Paige Nong, David A. Hanauer, Andrew Rosenberg, Jodyn Platt

**Affiliations:** 1 Department of Anesthesiology, University of Michigan, Ann Arbor, MI USA; 2 Department of Anesthesiology, Weill Cornell Medicine, New York, NY USA; 3 Division of Health Policy and Management, School of Public Health, University of Minnesota, Minneapolis, MN USA; 4 Department of Learning Health Sciences, University of Michigan, Ann Arbor, MI USA

**Keywords:** Computational models, Machine learning, Predictive medicine, Health care, Health care economics

## Abstract

Application of large language models in healthcare continues to expand, specifically for medical free-text classification tasks. While foundation models like those from ChatGPT show potential, alternative models demonstrate superior accuracy and lower costs. This study underscores significant challenges, including computational costs and model reliability. Amidst rising healthcare expenditures and AI’s perceived potential to reduce costs, a combination of local and commercial models might offer balanced solutions for healthcare systems.

## Introduction

Foundation artificial intelligence models, specifically large language models (LLMs), are increasingly used in clinical care and healthcare operations, though significant risks and challenges remain, including accountability, liability, ethical/legal use, bias, and equality as major concerns^1–3^. Without clinical validation of specific applications, healthcare systems and the patients they serve face substantial risk. Embedded in these concerns are the reliability of models and the financial costs required for operation at scale^4^, which we investigate in this report.

Increased accessibility to foundation models, such as OpenAI’s ChatGPT^5^, has caused widespread interest in LLMs to optimize healthcare operations, and specifically medical free-text classification, which covers a broad array of healthcare-related tasks. In revenue cycle operations, models are being used by payer systems to auto-process denials at low cost and high-throughput, further overloading the highly manual efforts of healthcare provider revenue cycle systems^6^. For providers, AI assistance deriving billing classifications from medical notes is new, but rapidly expanding, with potential positive returns for healthcare organizations if reliably executed^7,8^. While LLM use in the revenue cycle is intriguing, their accuracy, efficacy, and cost have not been rigorously compared to conventional machine learning (ML) approaches, which have demonstrated early success^9,10^.

Our goal was to provide insight into the question facing many healthcare organizations about whether to buy off-the-shelf AI tools versus creating their own based on performance and cost considerations. We investigate LLM execution time, accuracy, and usage costs in medical revenue cycle free-text classification tasks and consider the balance of commercial generalist versus locally developed specialist model capabilities and costs. Specifically, we explored the use of ChatGPT-4 for the classification of the 10th revision of the International Classification of Diseases (ICD) codes from electronic health records (EHR). This task represents a core challenge in billing and a potential strength of LLMs: mapping unstructured free-text to highly specific labels within a vast and complex solution space. Accurate classifications of billing codes directly impact reimbursement and reduce denials, underpayment, and audits. Furthermore, we have internally developed and deployed deep learning models for each ICD code classification grouping using Clinical-BigBird, a transformer framework, offering a readily available comparison^11,12^. We compared ChatGPT-4 to internal models in the separate classification of heart failure (HF) and chronic kidney disease (CKD) (Table 1). From 2999 notes, 1079 contained CKD ICD codes, 961 HF, and 930 neither HF nor CKD. Thirty were removed, containing both CKD and HF codes, yielding a final dataset of 2970 clinical notes from 191 unique patients. Notes with CKD or neither CKD/HF (*n* = 2009) were used to evaluate CKD, while HF + neither notes (*n* = 1891) were used to evaluate HF models. Two distinct Clinical-BigBird models were trained for either HF or CKD prediction, while two separate GPT-4 prompts were evaluated on ICD-labeling accuracy, F1 score, precision, recall, area under the receiver operating characteristic curve (ROC AUC), area under the precision-recall curve (PR AUC), and execution time. Table 1 shows lower accuracies (89.0% vs. 95.1%, CKD; 75.4% vs. 94.7%, HF), lower F1 scores (90.2% vs. 95.5%, CKD; 79.3% vs. 94.7%, HF), and longer execution times (2 minutes vs. 4 hours, CKD; 2 minutes vs. 6 hours, HF) for GPT-4 relative to Clinical-BigBird. GPT-4 models displayed higher false positive labeling than Clinical-BigBird (Supplemental Fig. 1). Slower GPT processing may be due to the computational execution for the LLM, data transfer, and latency, all of which are minimized using local models.

**Table 1:** Model results comparing Clinical-BigBird (BigBird) models and GPT-4, classifying chronic kidney disease (CKD), or heart failure (HF) from clinical notes

ICD class	Notes	Model	Acc	F1 Score	Precision	Recall	ROC AUC	PR AUC	Execution time
CKD	2009	BigBird	95.1%	95.5%	94.4%	96.6%	0.9924	0.7400	2 minutes
		GPT-4	89.0%	90.2%	86.8%	93.8%	0.9083	0.7500	4 hours
HF	1891	BigBird	94.7%	94.7%	96.4%	93.0%	0.9920	0.7400	2 minutes
		GPT-4	75.4%	79.3%	69.2%	92.8%	0.8135	0.6200	6 hours

Execution time is the time from submission of inputs to the model to the time of response (completion/output). Notes are the number of notes processed through each model. Abbreviations: Accuracy (Acc), Area Under the Receiver Operating Characteristic curve (ROC AUC), Area Under the Precision Recall curve (PR AUC).

LLMs from commercial vendors have pass-through costs (usage-based API billing costs from commercial LLM vendors) not incurred by internally developed models. In a second analysis, we utilized existing internal ML revenue cycle models to estimate pass-through costs for theoretical LLM conversion. We used token counts and cost estimates in four billing areas with existing non-LLM ML models in operation at our institution: (1) prior authorization of operating room surgical procedures (Prior Auth), (2) anesthesia and surgical final billing (Anes+Sx), (3) ICD classification of diseases (ICD), and (4) medical procedure unit prior authorization (MPU). These models were created using various non-LLM ML techniques and each used clinical and operative notes and included variation in daily average notes processed (300–2200), classification groups per billing area (25–1000, representing distinct models classifying distinct billing code bundles), input (prompt + input clinical text, 2195–3365) and completion (output text, 100) tokens (fundamental units of text and the method of pricing for LLM pass-through costs (Table 2). Tokens can represent whole or parts of words, or characters, depending on the tokenization method. As pricing models fluctuate over time, we used publicly available GPT pricing at the time of writing to represent the most recent, and hence most favorable, high and low-cost estimates: (GPT 4.1, $2/1 M input and $8/1 M output tokens; and GPT 4.1-nano batch pricing, $0.05/1 M input and $0.20/1 M output tokens, https://openai.com/api/pricing/, accessed 08/06/25). These calculations resulted in a wide representative range of estimated annual pass-through costs of $115k–$4.6 M, and illustrate potential costs incurred from running LLMs at scale.

**Table 2:** Large language model pricing estimations

Billing area	Daily average notes processed per classification group	Classification groups per billing area	Average tokens per note (input)	Average tokens per note (completion)	*Yearly costs (USD)	^Lowest Yearly Costs (USD)
Prior Auth	500	200	2195	100	$130,269	$3257
Anes+Sx	1000	200	2715	100	$312,746	$7819
ICD	2200	1000	3365	100	$4,158,066	$103,952
MPU	300	25	2520	100	$10,994	$275
				TOTAL	$4,612,075	$115,302

*Yearly costs are calculated using the pricing of the latest GPT model at the time of writing (08/06/25), GPT-4.1, with input and completion costs per 1 M tokens of $2 and $8, respectively. ^Lowest yearly costs based on the lowest latest GPT model pricing at time of writing (08/06/25), GPT 4.1-nano batch pricing with input and completion costs per 1 M tokens of $0.05 and $0.20, respectively. All other values are derived from the current revenue cycle AI operations at the institution.

These two separate analyses show that local models created using Clinical-BigBird perform better (Table 1) and are relatively less costly than LLMs (Table 2). However, costs for development and maintenance of AI systems can be significant for healthcare organizations, which should be considered^13^. Developing and maintaining models at enterprise health system scales have economic and computational challenges, including alignment of local information technology, health service, and computational specialists. Additionally, Health Insurance Portability and Accountability Act (HIPAA)-compliant infrastructure and high-level support are required for security. As with any AI use, liability falls on the end user and hospital systems that employ them. Healthcare compliance risks loom large, and the majority of intended uses are designed with human-in-the-loop. To minimize data leakage, local information assurance is critical, and running all models on local cloud infrastructure is the optimized strategy. External cloud connections with commercial LLMs contain compliance risks and data egress issues, significantly increasing the risk of data exposure.

Development of in-house, de-novo healthcare-specific LLMs would require enormous datasets across various institutions, computer science and data engineers, dedicated medical coders, hardware, and dedicated development and operations teams. Individuals can have payroll costs exceeding $100k/year, on-prem graphic processing unit (GPU) server costs can exceed $200k, and cloud GPU services per-hour pricing can be upwards of $40/hour (320 or 640 GiB GPU memory)^14^. While estimates are variable and difficult to predict, internal development to support these capabilities is limited to healthcare organizations with high-level resources^15^. Safety net organizations, for example, lack the capacity and workforce to implement high-cost systems^16^. While commercial LLMs still require infrastructure and support, these may be lighter requirements. Even considering data ingress, egress, storage, and maintenance costs, on top of vendor pricing ranging from $10–100k annually per solution^17^, commercial LLMs may be substantially cheaper and bear less failure risk than in-house development.

Pass-through costs are large but can be decreased by reducing the number of model passes through pre-classification of code groups and ensemble methods to batch the process into focused tasks. In our revenue cycle examples, notes could be pre-classified into surgical specialties and subsequently sent to specialty models only, reducing overall costs by using domain-sensitive LLMs to analyze specific notes. Another method to reduce costs is to reduce the prompt size. The prompts used in our investigations were large, representing a prompt: note ratio of 1.02:1 for CKD and 4.28:1 for HF. While attempts to reduce the prompt length resulted in worse performance, prompt engineering methods need to be explored, as there is an opportunity to further reduce costs and improve performance.

While LLMs performed relatively well in our investigations with minimal reconfiguration, results were worse than local models constructed for individual tasks, coinciding with previous studies. Investigation of task sizes and configurations found LLM performance to deteriorate as the number of questions, prompt sizes, and number of notes increased^18^. High-capacity (larger) models performed better, but still deteriorated around 50 tasks. For revenue cycle operations, LLMs have performed poorly. For ICD code classification, studies found low levels of agreement with human billers (10–25%) and a high level of hallucinations (35%)^19,20^. CPT code classification was also poor using LLMs—correctly identifying these codes in only ~35% of endovascular neurosurgical procedures^21^. Another study classifying both ICD and CPT codes found the highest matched rate to be <50% (GPT-4) and deemed “LLMs not appropriate for use on medical coding tasks” with LLMs “often generating codes conveying imprecise or fabricated information.”^22^ Another study found neither ChatGPT nor Gemini correctly identified both the CPT code and modifier for any case, with both models producing multiple responses with partially or completely inaccurate codes^23^. Our comparison with ICD codes performed better than previous studies, likely due to a focus on a smaller coding subset, but even with this subset, LLMs significantly underperformed Clinical-BigBird. Another consideration is keeping “up-to-date” information. Medical knowledge is vast, complex, and constantly evolving. LLMs have opaque training sets and unclear access to up-to-date medical research and guidelines, jeopardizing their utility in clinical tasks, favoring locally tuned, potentially lighter-weight models to achieve specific tasks. LLMs fine-tuned on domain-specific data, or enhanced through prompt engineering and in-context learning, have performed better than local deep learning models in some scenarios^24,25^. As an alternative to the Clinical-BigBird framework explored in this study, lightweight, extensible frameworks for running language models on local machines (e.g., Ollama, https://ollama.com/; gpt-oss-120b and gpt-oss-20b, https://openai.com/open-models/) and LLM fine-tuning should be further explored. However, fine-tuning or adapting large commercial LLMs to clinical tasks is non-trivial, comes with additional costs, and may not have permissions for use with sensitive data. Fine-tuning LLMs with medical data can expose the training data, and a balance of the benefits of fine-tuning to the protection of sensitive patient data is warranted^26^. Furthermore, our results warrant further investigation into alternative state-of-the-art foundation models such as Gemini (Google), Claude (Anthropic), and DeepSeek (DeepSeek), which could offer competitive performance and/or cost advantages. Finally, future studies should examine additional clinical conditions to expand the generalizability of this work and identify variation.

The main contributions of this communication prove the utility of AI in hospital revenue cycle operations and provide valuable insight into the costs using locally developed models vs. LLMs. Untuned LLMs show promise and offer convenience but require additional work to reach their full utility. Everyone is hoping that as functionality improves and costs reduce, they can be important technologies to solve healthcare problems. Recent surveys show high healthcare interest and perceived need for AI solutions, with 94% of C-suite executives recognizing AI as crucial to their success in the next 5 years^27^. This is due to the perceived notion that AI applications can reduce labor costs. With hospitals spending 56% of their total operating revenue on labor^28^, U.S. Healthcare spending grew 7.5% in 2023, reaching an astounding 17.6% of the nation’s Gross Domestic Product^29^. AI solutions may not only be an interest but a necessity. AI’s integration into healthcare is inevitable. While technical capabilities vary across different solutions, LLMs present a powerful opportunity to accelerate adoption, especially if quality improves and costs are reduced. While a combination of local and vendor solutions is likely optimal for most medical centers, those with adequate resources may currently favor local solutions for reduced pricing, ease of development, and ability for customization.

## Methods

### Study design and data

This study is a retrospective cohort review of EHR records from a single major academic tertiary care hospital (University of Michigan) cardiology and nephrology clinics, between 1 January 2013, and 31 December 2023, representing the same dataset used to train and test the Clinical-BigBird models. Clinical free-text from the EHR was used as the input for each model, as the primary source of manual revenue cycle code assignments. All data were extracted from the EHR system and were reviewed and approved by the University of Michigan’s Institutional Review Board (HUM00203986), and the authors followed STROBE guidelines^30^. A random set of 2999 clinical notes was collected from outpatient nephrology, family medicine, and cardiology clinics at the University of Michigan between 1/7/2013 and 6/14/2023 (Supplemental Table 1). There was a 3000-note limitation for the use of LLMs from our internal information assurance team due to the sensitive PHI contained in the notes.

### Models

We engineered prompts to create two distinct ChatGPT-4 models for classifying CKD and HF ICD code bundles from clinical notes (Supplemental). Prompt engineering was an iterative process, resulting in long, detailed prompts yielding the highest accuracies. We compared these models to internally developed ML models using Clinical-BigBird, each in operation at our healthcare institution at the time of preparing this manuscript. We evaluated these models on accuracy, F1 score, area under the receiver operating characteristic curve (ROC AUC), area under the precision-recall curve (PR AUC), and execution time. ICD accuracy was defined as correct classification of the bundle (i.e., was one of the ICD codes for heart failure predicted when one of the codes was assigned by billing teams for each note, the specific ICD codes did not need to match). ChatGPT-4 was selected for comparison as the most powerful GPT release at the time of the study. This model was accessed via API, privately hosted on a local HIPAA-secure Azure cloud infrastructure, and was available through an OpenAI (San Francisco, CA, USA)/Microsoft (Redmond, WA, USA) enterprise license through the University of Michigan. Prompt iteration and manual review were used for GPT prompt engineering.

Two separate Clinical-BigBird models were created from EHR data(Supplemental Table 1). One for chronic kidney disease using 149,702 unique clinical notes from outpatient family medicine and nephrology specialty clinics between 7/1/2013 and 7/1/2023. A second model was created for heart failure using 94,965 unique clinical notes from outpatient cardiology specialty clinics between 1/1/2013 and 1/1/2023. The training process begins with text preprocessing, including removal of common and infrequent words, stop words, and summarization to obtain the training input sequences. A pipeline was created to initialize the Clinical-BigBird model, configured for single-label classification, along with the optimizer and scheduler to achieve a global optimum. The model is provided with a dictionary of unique labels and their IDs, after which training sequences and true labels are fed into the pipeline. Predictions are compared against true labels (ex., HF or not), and cross-entropy loss is applied to evaluate distance from truth, with loss and accuracy recorded throughout training. With data imbalance, the loss function may be adjusted with class weights or dataset down-sampling. Analyzing loss and accuracy plots, along with prediction analysis, informed adjustments to the optimizer scheduler, data cleaning, or other parameters to refine the model. For evaluation, the AUC-ROC score was used, given the binary classification and data imbalance, and the confusion matrix was continuously reviewed for predictions, engaging stakeholders for verification. Iterations to improve results involved adjusting factors as needed, either one at a time or simultaneously.

In a second analysis, we examined annual pass-through costs if GPT models replaced local Clinical-BigBird models in four billing areas with AI classification models currently in operations at our institution: (1) prior authorization of operating room surgical procedures (Prior Auth), (2) anesthesia and surgical final billing predictions (Anes+Sx), (3) ICD classification of diseases from clinical notes (ICD), and (4) MPU. Between 1 January 2024, and 31 December 2024, for each billing area, we derived the daily average notes processed per classification group, the classification groups per billing area (number of models), and average tokens per note for prompt and completion. Yearly costs were derived from this data and publicly available ChatGPT pricing to complete a comprehensive cost analysis of existing revenue cycle AI models.

### HF and CKD classification—text preprocessing and labeling

Clinical notes were limited to the following types: Progress Note, History and Physical, Procedure Note, or Operative Note. Each note was preprocessed as follows: removed all words to the end of a sentence (existence of any character which is not a white space nor an alphabet) following a negation word and derivatives (not, no, neither, without, doesn’t, isn’t, hasn’t, didn’t), remove dates, special characters, numbers, units of measure, extra spaces, common stop words, and uncommon words from the training dataset (words used <5 times in total). Term frequency-inverse document frequency was used to reduce the text to the most important 500 words. Following preprocessing, each note was input into the ML models. Human medical coders were used as the reference group and source of performance comparison. HF positive were clinical notes with at least one of the following ICD codes manually assigned by the billing team: I50.1, I50.20, I50.21, I50.22, I50.23, I50.30, I50.31, I50.32, I50.33, I50.40, I50.41, I50.42, I50.43, I50.9, I50.810, I50.811, I50.812, I50.813, I50.814, I50.82, I50.83, I50.84, I50.89. CKD positive cases were one or more ICD codes starting with N18.

### Cost and time analyses

Four existing local AI billing areas were identified for cost evaluations: prior Auth, Anes+Sx, ICD, MPU. For their operational use, Prior Auth models use orders, case requests, and clinical notes as inputs; Anes+Sx models use brief and full operative and clinical notes; ICD models use all clinical notes up to 1 year before the encounter; MPU models use order sets and clinical notes. These models produce billing codes as outputs (ICD and CPT codes) and were developed using various non-LLM ML modeling methods. Yearly costs were derived from this data and publicly available ChatGPT pricing (“pass-through” costs, API pricing, https://openai.com/api/pricing/) to complete a comprehensive cost analysis of existing revenue cycle AI models. Execution time was calculated using the script/program running time for each model. The code recorded the start time and end time, while the total execution time was derived by calculating the difference. GPT-4 was used in the accuracy and execution time comparisons with Clinical-BigBird. We used the latest model pricing to represent API costs of the GPT models available (GPT 4.1, 08/06/2025, input and completion cost per 1 M token pricing of $2 and $8, respectively). GPT 4.1-nano was unavailable at the time of the study, but batch pricing for this model was used for comparison, representing the lowest costs of the GPT models available (input and completion cost per 1 M token pricing of $0.05 and $0.20, respectively). Costs were calculated by multiplying the prompt token and completion token by input and output pricing, respectively. Summing up those values provides an average per-note cost for each billing area. Multiplying by the number of notes and the number of classification groups for each area gives the yearly costs.

## Supplementary information

Supplemental

## Data Availability

The datasets generated and/or analyzed during the current study are not publicly available as they are defined as limited datasets per United States Federal Regulations and require execution of a data use agreement for transfer or use of the data. The investigative team is able to share data securely and transparently conditionally on: (i) receipt of a detailed written request identifying the requestor, purpose and proposed use of the shared data, (ii) use of a secure enclave for sharing of personally identifiable information and (iii) the request is permissible within the confines of existing data use agreements.
