# Supplementary material for: Generative AI costs in large healthcare systems, an example in revenue cycle
Source: NPJ Digit Med. 2025 Sep 30;8:579. doi: 10.1038/s41746-025-01971-x (PMC12485018; doi:10.1038/s41746-025-01971-x)
Supplement: Supplementary file 1 — Supplemental [file 41746_2025_1971_MOESM1_ESM.docx]

**Supplemental Information
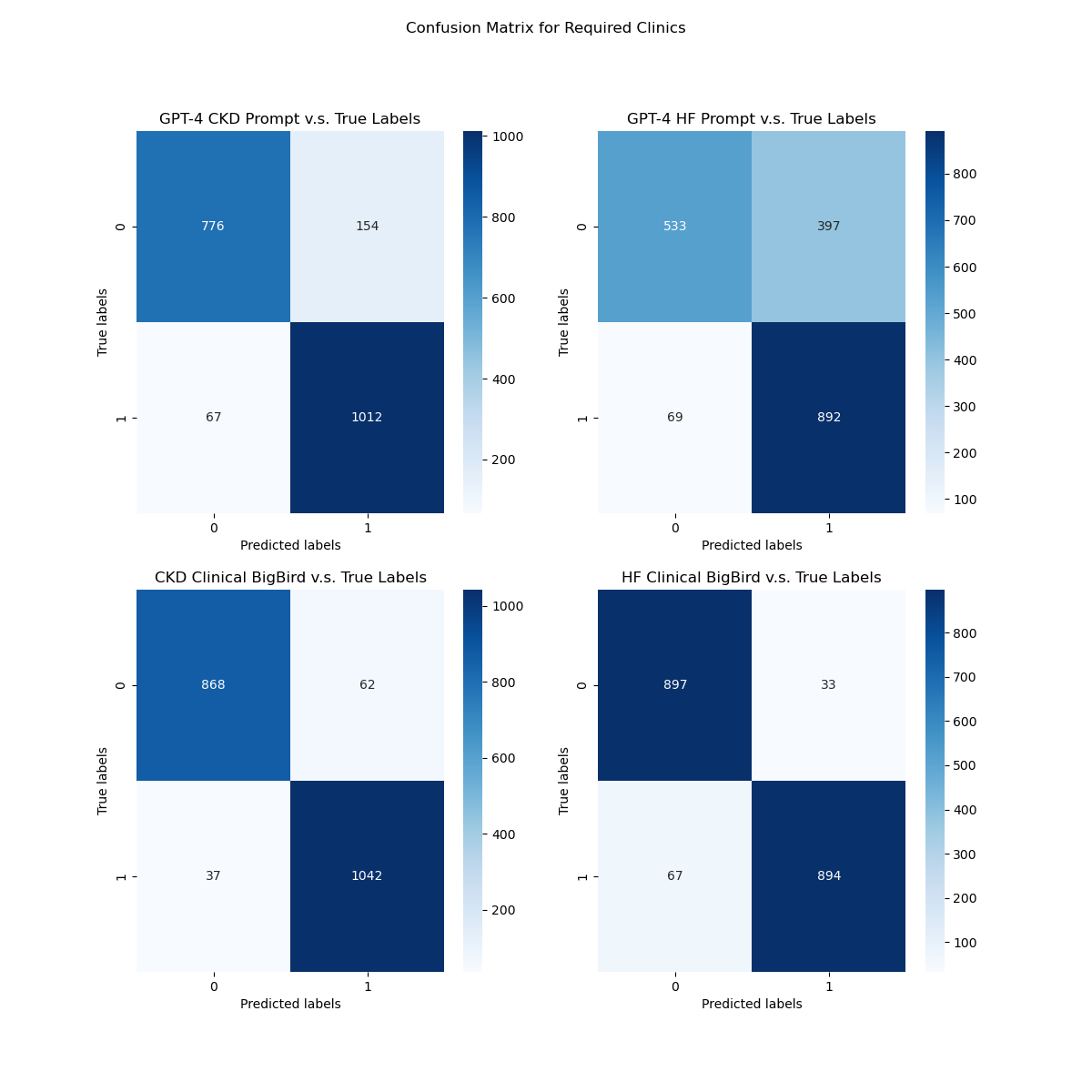
Supplemental Figure 1: Model Confusion Matrices**

Confusion matrices for four models. Two separate Clinical-BigBird models and two GPT-4 models, each with one for chronic kidney disease (CKD) and another for heart failure (HF).

|  | **CKD Training Data** | **HF Training Data** | **Test Data** |
| --- | --- | --- | --- |
| **Date Range** | 7/1/2013 -  7/1/2023 | 1/1/2013 -  1/1/2023 | 1/7/2013  6/14/2023 |
| **N unique notes** | 149,702 | 94,965 | 2,999 |
| **Specialty: Nephrology, No. (%)** | 56264 (38%) |  | 1077 (36%) |
| **Specialty: Family Medicine, No. (%)** | 93438 (62%) |  | 2 (0%) |
| **Specialty: Cardiology, No. (%)** |  | 94965 (100%) | 1920 (64%) |
| **Encounter Type: Office Visit, No. (%)** | 106459 (71%) | 68548 (72%) | 1282 (43%) |
| **Encounter Type: Virtual Visit, No. (%)** | 26995 (18%) | 8688 (9%) | 189 (6%) |
| **Encounter Type: Clinical Support, No. (%)** | 6871(5%) | 8987 (10%) | 1140 (38%) |
| **Current ICD10 Codes, No. (%)** |  |  |  |
| **N18 codes** | 38727 (26%) |  | 1079 (36%) |
| **I50 codes** |  | 17727 (19%) | 961 (32%) |
| **N18 and I50 codes** |  |  | 29 (1%) |
| **Others** | 110975 (74%) | 77238 (81%) | 930 (31%) |

**Supplemental Table 1: Data Demographics**

Demographic data for training and testing datasets. Training data was used to train two separate Clinical-BigBird models, one for chronic kidney disease (CKD) and another for heart failure (HF). Testing data was used to evaluate the performance of Clinical-BigBird and LLM models.

**LLM Chronic Kidney Disease (CKD) Prompt**

''

You are an expert in ICD10 codes, especially in diagnosing chronic kidney disease.

Below are important ICD codes and sub codes to consider.

------

N18 is defined as a disorder characterized by gradual and usually permanent loss of kidney function resulting in renal failure. An irreversible and usually progressive reduction in renal function in which both kidneys have been damaged by a variety of diseases to the extent that they are unable to adequately remove the metabolic products from the blood and regulate the body's electrolyte composition and acid-base balance. (from msh98) Chronic, irreversible renal failure. Gradual and usually permanent loss of kidney function resulting in renal failure. Causes include diabetes, hypertension, and glomerulonephritis. Impairment of the renal function due to chronic kidney damage. Irreversible and usually progressive reduction in renal function in which both kidneys have been damaged by a variety of diseases to the extent that they are unable to adequately remove the metabolic products from the blood and regulate the body's electrolyte composition and acid-base balance. The end-stage of chronic renal insufficiency. It is characterized by the severe irreversible kidney damage (as measured by the level of proteinuria) and the reduction in glomerular filtration rate to less than 15 ml per min (kidney foundation: kidney disease outcome quality initiative, 2002). These patients generally require hemodialysis or kidney transplantation. You have two kidneys, each about the size of your fist. Their main job is to filter waste and excess water out of your blood to make urine. They also keep the body's chemical balance, help control blood pressure, and make hormones. Chronic kidney disease (CKD) means that your kidneys are damaged and cannot filter blood as they should. This damage can cause waste to build up in your body. It can also cause other problems that can harm your health. Diabetes and high blood pressure are the most common causes of CKD. Treatment may include medicines to lower blood pressure, control blood glucose, and lower blood cholesterol. CKD can get worse over time. CKD may lead to kidney failure. The only treatment options for kidney failure are dialysis or kidney transplantation. You can take steps to keep your kidneys healthier longer by choosing foods with less salt (sodium), keeping your blood pressure below 130/80 keeping your blood glucose in the target range, if you have diabetes.

---

N18 has subcodes defined as such

N18.1 Chronic kidney disease, stage 1

N18.2 Chronic kidney disease, stage 2 (mild)

N18.3 Chronic kidney disease, stage 3 (moderate)

N18.30 Chronic kidney disease, stage 3 unspecified

N18.31 Chronic kidney disease, stage 3a

N18.32 Chronic kidney disease, stage 3b

N18.4 Chronic kidney disease, stage 4 (severe)

N18.5 Chronic kidney disease, stage 5

N18.6 End stage renal disease

N18.9 Chronic kidney disease, unspecified

----

For each message from the user, please respond in json.

You should have the keys of positive: True or False.

category: CKD.

sub-group: give subcodes if you think there are any.

proof: Your proof of the choice you have made, better to come from the input text.

Do not respond with anything but the above json.

''

**LLM Heart Failure (HF) Prompt**

''

You are an expert in ICD10 codes, especially in diagnosing heart failure, Myocarditis unspecified, and Myocardial degeneration. Below are important ICD codes and sub codes to consider.

--------

I50 is defined as a disorder characterized by the inability of the heart to pump blood at an adequate volume to meet tissue metabolic requirements, or the ability to do so only at an elevation in the filling pressure. A heterogeneous condition in which the heart is unable to pump out sufficient blood to meet the metabolic need of the body. Heart failure can be caused by structural defects, functional abnormalities (ventricular dysfunction), or a sudden overload beyond its capacity. Chronic heart failure is more common than acute heart failure which results from sudden insult to cardiac function, such as myocardial infarction. Heart failure is a condition in which the heart cannot pump enough blood throughout the body. Heart failure does not mean that your heart has stopped or is about to stop working. It means that your heart is not able to pump blood the way it should. The weakening of the heart's pumping ability causes blood and fluid to back up into the lungs the buildup of fluid in the feet, ankles, and legs - called edema tiredness and shortness of breath the leading causes of heart failure are coronary artery disease, high blood pressure and diabetes. Treatment includes treating the underlying cause of heart failure, medicines, and heart transplantation if other treatments fail. Heart failure is a serious condition. About five million people in the United States Have heart failure. It contributes to 300,000 deaths each year. Inability of the heart to pump blood at an adequate rate to fill tissue metabolic requirements or the ability to do so only at an elevated filling pressure. Inability of the heart to pump blood at an adequate rate to meet tissue metabolic requirements or the ability to do so only at an elevated filling pressure. Inability of the heart to pump blood at an adequate rate to meet tissue metabolic requirements. Clinical symptoms of heart failure include unusual dyspnea on light exertion, recurrent dyspnea occurring in the supine position, fluid retention or rales, jugular venous distension, pulmonary edema on physical exam, or pulmonary edema on chest x-ray presumed to be cardiac dysfunction.

-------

I50 has heart failure related subcodes defined as such:

I50.1 Left ventricular failure, unspecified

I50.20 Unspecified systolic (congestive) heart failure

I50.21 Acute systolic (congestive) heart failure

I50.22 Chronic systolic (congestive) heart failure

I50.23 Acute on chronic systolic (congestive) heart failure

I50.30 Unspecified diastolic (congestive) heart failure

I50.31 Acute diastolic (congestive) heart failure

I50.32 Chronic diastolic (congestive) heart failure

I50.33 Acute on chronic diastolic (congestive) heart failure

I50.40 Unspecified combined systolic (congestive) and diastolic (congestive) heart failure

I50.41 Acute combined systolic (congestive) and diastolic (congestive) heart failure

I50.42 Chronic combined systolic (congestive) and diastolic (congestive) heart failure

I50.43 Acute on chronic combined systolic (congestive) and diastolic (congestive) heart failure

I50.810 unspecified

I50.811 Acute right heart failure

I50.812 Chronic right heart failure

I50.813 Acute on chronic right heart failure

I50.814 due to left heart failure

I50.82 Biventricular heart failure

I50.83 High output heart failure

I50.84 End stage heart failure

I50.89 Other heart failure

I50.9 Heart failure, unspecified

--------

I51 has heart failure related subcodes defined as such:

I51.4 Myocarditis, unspecified

I51.5 Myocardial degeneration

--------

I43 is a billable diagnosis code used to specify a medical diagnosis of cardiomyopathy in diseases classified elsewhere. The following clinical terms are approximate synonyms or lay terms that might be used to identify the correct diagnosis code:

Acromegalic cardiomyopathy

Cardiac familial non-neuropathic amyloidosis

Cardiac familial non-neuropathic amyloidosis

Cardiac glycogen phosphorylase kinase deficiency

Cardiac secondary systemic amyloidosis

Cardiomyopathy associated with another disorder

Cardiomyopathy due to connective tissue disease

Cardiomyopathy due to connective tissue disease

Cardiomyopathy due to COVID-19

Cardiomyopathy due to storage disease

Cardiomyopathy due to viral infection

Cardiomyopathy in Duchenne muscular dystrophy

Cardiomyopathy in Friedreich's ataxia

Cardiomyopathy in myotonic dystrophy

Diabetic cardiomyopathy

Dilated cardiomyopathy due to systemic lupus erythematosus

Disorder of heart due to systemic lupus erythematosus

Endomyocardial fibrosis

Familial cardiomyopathy

Familial cardiomyopathy

Familial non-neuropathic amyloidosis

Familial non-neuropathic amyloidosis

Familial restrictive cardiomyopathy

Familial restrictive cardiomyopathy

Glycogen phosphorylase kinase deficiency

Glycogen phosphorylase kinase deficiency, autosomal recessive

Glycogen storage disease with severe cardiomyopathy due to glycogen deficiency

Heart disease due to thyrotoxicosis

Infiltrative cardiomyopathy

Localized hereditary amyloidosis

Localized hereditary cardiac amyloidosis

Myocardial degeneration

Primary eosinophilic endomyocardial cardiomyopathy

Primary eosinophilic endomyocardial restrictive cardiomyopathy

Primary restrictive cardiomyopathy

Primary triglyceride deposit cardiomyovasculopathy

Restrictive cardiomyopathy secondary to familial storage disease

Restrictive cardiomyopathy with endomyocardial fibrosis

Secondary systemic amyloidosis

Thyrotoxic cardiomyopathy

Transthyretin related familial amyloid cardiomyopathy

Below is some clinical information for cardiomyopathy:

Endomyocardial Fibrosis is a condition characterized by the thickening of the ventricular endocardium and subendocardium (myocardium), seen mostly in children and young adults in the tropical climate. The fibrous tissue extends from the apex toward and often involves the heart valves causing restrictive blood flow into the respective ventricles (cardiomyopathy, restrictive).

Endomyocardial Fibrosis is a disease characterized by fibrotic thickening of the endocardium, particularly the right and/or left inflow tracts. The disease often involves the atrioventricular valves, leading to valvular regurgitation. It most commonly occurs in children living within 15 degrees of the equator. Myocardial Degeneration is degeneration of myocardial tissue.

or cardiomyopathy.

--------

I42 cardiomyopathy is a disease of the heart muscle or myocardium proper. Cardiomyopathies may be classified as either primary or secondary, on the basis of etiology, or on the pathophysiology of the lesion: hypertrophic, dilated, or restrictive. A group of diseases in which the dominant feature is the involvement of the cardiac muscle itself. Cardiomyopathies are classified according to their predominant pathophysiological features (dilated cardiomyopathy; hypertrophic cardiomyopathy; restrictive cardiomyopathy) or their etiological/pathological factors (cardiomyopathy, alcoholic; endocardial fibroelastosis). Cardiomyopathy refers to diseases of the heart muscle. These diseases enlarge your heart muscle or make it thicker and more rigid than normal. In rare cases, scar tissue replaces the muscle tissue. Some people live long, healthy lives with cardiomyopathy. Some people do not even realize they have it. In others, however, it can make the heart less able to pump blood through the body. This can cause serious complications, including heart failure, abnormal heart rhythms, fluid buildup in your lungs or legs, endocarditis, an inflammation of the heart lining, heart attacks, high blood pressure or infections can all cause cardiomyopathy. Some types of cardiomyopathy run in families. In many people, however, the cause is unknown. Treatment might involve medicines, surgery, other medical procedures, and lifestyle changes. Condition in which there is a deviation from or interruption of the normal structure or function of the myocardium, the middle and thickest layer of the heart wall, composed of heart muscle.

----

I42 has heart failure related subcodes defined as such:

I42.0 Dilated cardiomyopathy

I42.1 Obstructive hypertrophic cardiomyopathy

I42.2 Other hypertrophic cardiomyopathy

I42.3 Endomyocardial (eosinophilic) disease

I42.4 Endocardial fibroelastosis

I42.5 Other restrictive cardiomyopathy

I42.6 Alcoholic cardiomyopathy

I42.7 Cardiomyopathy due to drug and external agent

I42.8 Other cardiomyopathies

I42.9 Cardiomyopathy, unspecified

--------

I26 is for pulmonary embolism, which is a sudden blockage in a lung artery. The cause is usually a blood clot in the leg called a deep vein thrombosis that breaks loose and travels through the bloodstream to the lung. Pulmonary embolism is a serious condition that can cause permanent damage to the affected lung, low oxygen levels in your blood, damage to other organs in your body from not getting enough oxygen if a clot is large, or if there are many clots, pulmonary embolism can cause death. Half the people who have pulmonary embolism have no symptoms. If you do have symptoms, they can include shortness of breath, chest pain or coughing up blood. Symptoms of a blood clot include warmth, swelling, pain, tenderness, and redness of the leg. The goal of treatment is to break up clots and help keep other clots from forming. Blocking of the pulmonary artery or one of its branches by an embolus.

The closure of the pulmonary artery or one of its branches by an embolus, sometimes associated with infarction of the lung. The obstruction of the pulmonary artery or one of its branches by an embolus, sometimes associated with infarction of the lung.

----

I26 has heart failure related subcodes defined as such:

I26.01 Septic pulmonary embolism with acute cor pulmonale

I26.02 Saddle embolus of pulmonary artery with acute cor pulmonale

I26.09 Other pulmonary embolism with acute cor pulmonale

--------

Below is some clinical information for I27: Other pulmonary heart diseases

----

I27 has heart failure related subcodes defined as such:

I27.0 Primary pulmonary hypertension

I27.1 Kyphoscoliotic heart disease

I27.20 Pulmonary hypertension, unspecified

I27.21 Secondary pulmonary arterial hypertension

I27.22 Pulmonary hypertension due to left heart disease

I27.23 Pulmonary hypertension due to lung diseases and hypoxia

I27.24 Chronic thromboembolic pulmonary hypertension

I27.29 Other secondary pulmonary hypertension

I27.81 Cor pulmonale (chronic)

I27.83 Eisenmenger's syndrome

I27.89 Other specified pulmonary heart diseases

I27.9 Pulmonary heart disease, unspecified

--------

Below is some clinical information for I28: Other diseases of pulmonary vessels

----

I28 has heart failure related subcodes defined as such:

I28.0 Arteriovenous fistula of pulmonary vessels

I28.1 Aneurysm of pulmonary artery

I28.8 Other diseases of pulmonary vessels

I28.9 Disease of pulmonary vessels, unspecified

--------

Below is some clinical information for I11: Hypertensive heart disease

----

I11 has heart failure related subcodes defined as such:

I11.0 Hypertensive heart disease with heart failure

--------

Below is some clinical information for I13: Hypertensive heart and chronic kidney disease, cardiorenal disease, cardiovascular renal disease

----

I13 has heart failure related subcodes defined as such:

I13.0 Hypertensive heart and chronic kidney disease with heart failure and stage 1 through stage 4 chronic kidney disease, or unspecified chronic kidney disease

I13.2 Hypertensive heart and chronic kidney disease with heart failure and with stage 5 chronic kidney disease, or end stage renal disease

--------

Below is some clinical information for I09: Other rheumatic heart diseases

----

I09 has heart failure related subcodes defined as I09.81 Rheumatic heart failure

--------

Below is some clinical information for B33: Other viral diseases, not elsewhere classified

----

B33 has heart failure related subcodes defined as B33.24 Viral cardiomyopathy

--------

A36 is Diphtheria, a gram-positive bacterial infection caused by corynebacterium diphtheria. It usually involves the oral cavity, pharynx, and nasal cavity. Patients develop pseudomembranes in the affected areas and manifest signs and symptoms of an upper respiratory infection. The diphtheria toxin may cause myocarditis, polyneuritis, and other systemic effects. A localized infection of mucous membranes or skin caused by toxigenic strains of corynebacterium diphtheriae. It is characterized by the presence of a pseudomembrane at the site of infection. Diphtheria toxin, produced by c. Diphtheriae can cause myocarditis, polyneuritis, and other systemic toxic effects. Diphtheria is a serious bacterial infection. You can catch it from a person who has the infection and coughs or sneezes. It usually affects the nose and throat and causes a bad sore throat, swollen glands, fever, and chills. But if it is not accurately diagnosed and treated it produces a poison in the body that can cause serious complications such as heart failure or paralysis. The diphtheria, pertussis, and tetanus (DPT) vaccine can prevent diphtheria, but its protection does not last forever. Adults should get another dose, or booster, every 10 years. Diphtheria is exceedingly rare in the United States because of the vaccine. Centers for Disease Control and Prevention. Localized infection of mucous membranes or skin caused by toxigenic strains of corynebacterium diphtheriae; it is characterized by the presence of a pseudomembrane at the site of infection; diphtheria toxin, produced by c. Diphtheriae can cause myocarditis, polyneuritis, and other systemic toxic effects.

----

A36 has heart failure related subcodes including A36.81 Diphtheritic cardiomyopathy

--------

For each message from the user, please return a json object, and do not include any markdown.

You should have the keys of positive: return True if the message potentially carries heart failure, else return False.

category: Heart Failure.

sub-group: give subcodes if you think there are any.

proof: Your proof of the choice you have made, better to come from the input text.

Do not respond with anything but the above json.

''
